# Principal component analysis for ataxic gait using a triaxial accelerometer

**DOI:** 10.1186/s12984-017-0249-7

**Published:** 2017-05-02

**Authors:** Akira Matsushima, Kunihiro Yoshida, Hirokazu Genno, Shu-ichi Ikeda

**Affiliations:** 1 Department of Neurology and Rheumatology, Shinshu University School of Medicine, Matsumoto, Japan; 2 JA Nagano Koseiren Kakeyu-Misayama Rehabilitation Center Kakeyu Hospital, Ueda, Japan; 3 Division of Neurogenetics, Department of Brain Disease Research, Shinshu University School of Medicine, Matsumoto, Japan; 4 Kissei Comtec Co., Ltd, Matsumoto, Japan

**Keywords:** Cerebellar ataxia, Triaxial accelerometer, Ataxic gait, Gait analysis, Principal component analysis, SARA

## Abstract

**Background:**

It is quite difficult to evaluate ataxic gait quantitatively in clinical practice. The aim of this study was to analyze the characteristics of ataxic gait using a triaxial accelerometer and to develop a novel biomarker of integrated gate parameters for ataxic gait.

**Methods:**

Sixty-one patients with spinocerebellar ataxia (SCA) or multiple system atrophy with predominant cerebellar ataxia (MSA-C) and 57 healthy control subjects were enrolled. The subjects were instructed to walk 10 m for a total of 12 times on a flat floor at their usual walking speed with a triaxial accelerometer attached to their back. Gait velocity, cadence, step length, step regularity, step symmetry, and degree of body sway were evaluated. Principal component analysis (PCA) was used to analyze the multivariate gait parameters. The Scale for the Assessment and Rating of Ataxia (SARA) was evaluated on the same day of the 10-m walk trial.

**Results:**

PCA divided the gait parameters into four principal components in the controls and into two principal components in the patients. The four principal components in the controls were similar to those found in earlier studies. The second principal component in the patients had relevant factor loading values for gait velocity, step length, regularity, and symmetry in addition to the degree of body sway in the medio-lateral direction. The second principal component score (PCS) in the patients was significantly correlated with disease duration and the SARA score of gait (*ρ* = −0.363, *p* = 0.004; *ρ* = −0.574, *p* < 0.001, respectively).

**Conclusions:**

PCA revealed the main component of ataxic gait. The PCS of the main component was significantly different between the patients and controls, and it was well correlated with disease duration and the SARA score of gait in the patients. We propose that this score provides a novel method to assess the severity of ataxic gait quantitatively using a triaxial accelerometer.

## Background

Spinocerebellar ataxia (SCA) and multiple system atrophy (MSA) are major neurodegenerative diseases mainly affecting the cerebellum and brainstem. SCA and MSA patients with predominant cerebellar ataxia (MSA-C) usually show ataxic gait as the initial and cardinal symptom [1, 2]. Clinical scales such as the Scale for the Assessment and Rating of Ataxia (SARA) or Unified Multiple System Atrophy Rating Scale are easy to administer and can be used to assess the severity of gait disturbance [3, 4]; however, these scales are mostly qualitative and ordinal. A triaxial accelerometer is a well-known device for measuring human movement [5]. The measured values obtained using a triaxial accelerometer are objective, and test-retest reliability has been validated [6]. Triaxial accelerometers have been applied to several movement disorders to evaluate the characteristics of motor symptoms and the effectiveness of treatment [7–9]. Human gait is a highly complicated and integrated bipedal locomotion activity that can be characterized by multivariate data; therefore, simpler and more comprehensive methods are needed for the interpretation of such data. Several of the gait parameters obtained using a triaxial accelerometer have been proven to be useful for the clinical assessment of ataxia in SCA and MSA-C patients [10, 11]; however, a method for the integrated analysis of various gait parameters directly reflecting ataxic gait has not yet been developed. Principal component analysis (PCA) is a statistical approach used to process complicated multivariate data. PCA can reasonably integrate multivariate data covering some principal components (PCs) and can provide continuous variables by calculating the principal component score (PCS), which reflects the aspect of the principal component. PCA has been used recently in patients with dementia and aged patients to reveal the main factors accounting for gait disturbance [12, 13].

The purpose of this study was to reveal the characteristics of ataxic gait and to develop a novel method for the quantitative assessment of ataxic gait in SCA and MSA-C patients. For this purpose, we applied PCA to analyze the multivariate gait parameters obtained using a triaxial accelerometer.

## Methods

### Subjects

Patients whose SARA score of gait was greater than six points or whose SARA score of stance was greater than three points were excluded because they could not complete the walking task using a triaxial accelerometer. Patients who had comorbid conditions that affect motor function, such as cerebrovascular or orthopedic disorders, were also excluded. After the application of the exclusion criteria, 61 patients clinically diagnosed with SCA or MSA-C and 57 control subjects without gait impairment were enrolled in this study. All patients could stand and walk by themselves, but some used a cane or walker to avoid falling. Fifteen patients (2 with SCA type 6 [SCA6], 8 with SCA31, and 5 with MSA-C) and 18 control subjects were measured twice within an approximately 6-month interval to evaluate chronological changes. When the subjects were recruited, they were provided with all necessary information about the study and informed consent was obtained from all subjects. This study was approved by the Ethics Committee of Shinshu University School of Medicine (No. 2667).

### Instrumentation and measurements

A triaxial accelerometer (Jukudai Mate; Kissei Comtec Co., Ltd., Matsumoto, Japan) was used. The device was small (size, 55 mm × 80 mm; thickness, 10 mm) and light (weight, ~90 g). It had a sampling rate of 20 Hz. The range of detection was between −10 G and +10 G (G: acceleration of gravity, 1 G = 9.80665 m/s^2^) and the resolution power was 0.02 G. The data acquired by the device were analyzed by BIMUTAS II (Kissei Comtec Co., Ltd., Matsumoto, Japan), which was developed for biological processing. The device was attached to the back (median of L3) of the subject by an elastic belt. The subjects were asked to walk on a flat floor at a speed they were comfortable with. The walking distance was 10 m; however, the subjects were instructed to stop walking at 3 m beyond the end of the walkway. The walking test was repeated 12 times (6-times reciprocating walk) consecutively. For subjects who felt it difficult to walk 12 times, the test was aborted at the end of the third reciprocating walk (i.e., they completed the 10-m walk 6 times).

### Parameters

The axes of the direction of acceleration were defined in three dimensions: anterior-posterior direction (AP), medio-lateral direction (ML), and vertical direction (VT). Gait velocity, cadence, step length, auto-correlation coefficient (AC) in each direction, and the root mean square (RMS) in each direction were taken as gait parameters. Velocity was calculated by using the time taken to complete each 10-m walking test, which was measured with a manual stopwatch. Cadence, defined as steps per minute, was calculated by dividing the steps counted from the acceleration data in the 10-m walking by walking time. Step length was calculated using velocity and cadence.

We used an auto-correlation method to calculate AC [14, 15]. To compare the similarity of certain two different acceleration data, a cross-correlation function was used. When the compared two acceleration data were identical, the function was specially called as auto-correlation function [14, 15]. Each value of the auto-correlation function is a correlation coefficient between the raw acceleration data and the data to be shifted at some sample point from the same raw acceleration data. When the shifted sample point was zero, the position was defined as the reference point. The first AC peak next to the reference point represented step regularity [14, 15], and the ratio of the first AC peak next to the reference point to the second peak represented step symmetry [14]. RMS represented the degree of sway during gait [15]. By eliminating the beginning and end of the test, we used the acceleration data from the middle 6.4 s to calculate AC and RMS with as many data points as possible for each subject. As a result, the same number (128) of data points was analyzed in all subjects. The mean value according to the number of measurements was used for gait analysis in all parameters.

### Statistical analysis

Statistical analysis was performed using SPSS for Windows (IBM Statistics 22). The assumption of a normal distribution was assessed by the Kolmogorov-Smirnov test. Between-group differences in age and gender were assessed using an independent *t*-test (continuous data) and a chi-square test (categorical data), respectively. Differences in gait parameters between both groups were examined with an independent *t*-test. The correlation between disease duration and gait parameters was assessed by Spearman’s rank correlation coefficient. To validate the reliability of the gait parameters, an intra-class correlation coefficient (ICC) was used. Specifically, to focus on the reliability of a single time measurement, ICC (1, 1) was used.

In PCA, all gait parameters were included and standardized to zero mean and unit variance before PCA was performed. Varimax rotation was used to derive orthogonal factor loading. PCs with eigenvalues greater than one were considered to be relevant. Factor loading values greater than 0.4 as the absolute value were considered relevant [16]. The first and second PCSs were calculated for each subject as the linear combination of the factor loading values in the patients and standardized gait parameters in each subject. The scores were standardized to zero mean and unit variance. For multiple comparisons of PCS divided according to the SARA score of gait, one way analysis of variance (ANOVA) was used. The Bonferroni correction was applied as a post hoc test. The chronological change of PCSs was assessed by a dependent *t*-test. The level of significance was set at *p* < 0.05 in all tests.

## Results

The characteristics of the subjects are shown in Table 1. There were no significant differences in gender and age distribution between the patients and controls (gender: *χ* ^*2*^ = 0.131, *p* = 0.71; age: *t* = −1.881, *p* = 0.08).

**Table 1:** Characteristics of the subjects

	Patients *n* = 61	Controls *n* = 57
	Mean ± SD (range)	Mean ± SD (range)
Male / female, n	32 / 29	28 / 29
Age, years	61.1 ± 10.7 (39–83)	56.7 ± 14.6 (27–85)
Disease duration, years	9.2 ± 7.8 (0–41)	.
SARA score (total)	11.8 ± 5.6 (1–23)	
SARA score (gait)	2.7 ± 1.3 (0–6)	
Disease subtype		
SCA1	1	
SCA2	1	
SCA3/MJD	2	
SCA6	13	
SCA31	16	
ADCA ^a^	9	
CCA	10	
MSA-C	9	

*Abbreviations*: *CCA* cortical cerebellar ataxia, *MJD* Machado-Joseph disease, *MSA-C* multiple system atrophy with predominant cerebellar ataxia, *SARA* Scale for the Assessment and Rating of Ataxia, *SCA* spinocerebellar ataxia, *SD* standard deviation

^a^ Family history was supportive of autosomal dominant cerebellar ataxia (ADCA), but genetic testing was not performed

### Gait assessment

The values of each gait parameter are shown in Table 2. All gait parameters, except for step symmetry, were significantly different between the patients and controls. RMSs for AP and VT were significantly lower, whereas that for ML was significantly higher in the patients than in the controls. The ICC (1, 1) values were approximately or greater than 0.6 for all parameters, except step symmetry, in both groups.

**Table 2:** Measurements of each gait parameter

Parameter	Patients		Controls		*t-*value	*p-*value
	Mean ± SD	ICC (1, 1) (95% CI)	Mean ± SD	ICC (1,1) (95% CI)		
Velocity (m/s)	0.96 ± 0.27 ^a^	0.96 (0.95–0.97)	1.34 ± 0.13	0.87 (0.82–0.91)	9.922	<0.001
Cadence (step/min)	112.1 ± 11.5 ^a^	0.68 (0.60–0.77)	116.9 ± 7.7	0.90 (0.86–0.93)	2.545	0.012
Step length (m)	0.51 ± 0.12 ^a^	0.91 (0.88–0.94)	0.69 ± 0.06	0.87 (0.83–0.91)	10.128	<0.001
Step regularity in AP	0.51 ± 0.14 ^a^	0.71 (0.62–0.79)	0.70 ± 0.09	0.59 (0.49–0.69)	9.098	<0.001
Step regularity in VT	0.48 ± 0.15 ^a^	0.74 (0.66–0.81)	0.70 ± 0.09	0.61 (0.52–0.71)	9.241	<0.001
Step symmetry in AP	0.78 ± 0.08	0.15 (0.09–0.24)	0.78 ± 0.05	0.13 (0.08–0.22)	0.514	0.609
Step symmetry in VT	0.78 ± 0.07	0.12 (0.07–0.20)	0.81 ± 0.06	0.18 (0.02–0.28)	1.873	0.064
RMS in AP (m/s^2^)	1.74 ± 0.57 ^a^	0.87 (0.82–0.91)	2.15 ± 0.34	0.83 (0.77–0.88)	4.442	<0.001
RMS in ML (m/s^2^)	1.81 ± 0.61 ^a^	0.90 (0.86–0.93)	1.67 ± 0.41	0.89 (0.85–0.93)	−2.088	0.039
RMS in VT (m/s^2^)	2.21 ± 0.81 ^a^	0.91 (0.88–0.94)	2.71 ± 0.55	0.88 (0.83–0.92)	3.656	<0.001

*Abbreviations*: *AP* anterior-posterior, *CI* confidence interval, *ICC* intra-class correlation coefficient, *ML* medio-lateral, *RMS* root mean square, *SD* standard deviation, *VT* vertical

^a^ Significantly different between the patients and controls

### PCA

Factor loading values and the proportion of the variance in the patients and controls are shown in Table 3. In the controls, four PCs (PC1, PC2, PC3, and PC4) were found to explain 79% of the variance. In PC1, velocity, step length, and RMS in all directions were relevant. In PC2, 3, and 4, cadence, step symmetry, and step regularity were relevant, respectively.

**Table 3:** Factor loading values and the proportion of the variance explained by principal component analysis

Parameter	Patients		Controls			
	PC1	PC2	PC1	PC2	PC3	PC4
Velocity	**0.811**	**0.518**	**0.872**	0.318	0.008	0.200
Cadence	**0.697**	0.303	0.172	**0.901**	0.176	0.040
Step length	**0.622**	**0.480**	**0.847**	−0.345	−0.132	0.184
Step regularity in AP	−0.058	**0.932**	0.042	−0.318	0.144	**0.826**
Step regularity in VT	0.159	**0.897**	0.071	0.295	0.003	**0.872**
Step symmetry in AP	−0.040	**0.762**	0.086	−0.029	**0.903**	0.027
Step symmetry in VT	0.101	**0.778**	−0.095	0.220	**0.782**	0.138
RMS in AP	**0.930**	−0.057	**0.859**	0.279	0.115	−0.045
RMS in ML	**0.774**	**−0.434**	**0.461**	−0.01	0.386	−0.283
RMS in VT	**0.916**	−0.146	**0.674**	**0.568**	−0.034	−0.114
Variance explained (%)	38.7	36.6	29.4	16.7	16.6	16.3

Factor loading values greater than 0.4 as the absolute value are in bold

*Abbreviations*: *AP* anterior-posterior, *ML* medio-lateral, *PC* principal component, *RMS* root mean square, *VT* vertical

In the patients, PC1 was similar to that in the controls, except for the involvement of cadence. However, the factor loading values in PC2 were very different. In particular, RMS in ML was relevant and had a negative value.

**Fig. 1:**
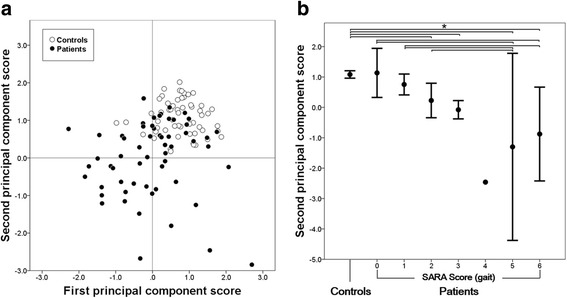
The distribution of the first and second principal component scores (PCSs). **a** Scatter diagram of the first and second PCSs in the patients and controls. *Both* scores were significantly higher in the controls than in the patients. **b** The distribution of the second PCS among the controls and the groups divided according to the SARA score of gait. The *bars* show the 95% confidence intervals. The number of the subjects in each group was 57 in controls, 61 in patients (2 with the SARA score of gait 0, 7 with score 1, 16 with score 2, 28 with score 3, 1 with score 4, 3 with score 5, and 4 with score 6). As there was only 1 subject in the patients with the score 4, the confidence interval in that group is not shown. *p < 0.05

The distributions of the first and second PCSs are shown in Fig. 1a. Both the first and second PCSs were significantly different between the patients and controls (*t* = 5.189, *p* < 0.001; *t* = 7.527, *p* < 0.001, respectively), and well correlated with disease duration and the SARA score of gait in the patients (*ρ* = −0.363, *p* = 0.004; *ρ* = −0.574, *p* < 0.001, respectively). The distributions of the second PCS in the patients and controls are shown in Fig. 1b. The second PCS was significantly different between the patients and controls. There was a significant difference between the subgroups (ANOVA: *F*_*(5, 53)*_ = 16.866, *p* < 0.001; post hoc test: between SARA score of gait 0 and that of 5 and 6; between that of gait 1 and that of 5 and 6; between that of 2 and that of 5). The ICC (1, 1) values of the first PCS were 0.92 (95% confidence interval [CI]: 0.89–0.95) in the patients and 0.85 (95% CI: 0.75–0.89) in the controls; those of the second PCS were 0.63 (95% CI: 0.54–0.72) in the patients and 0.33 (95% CI: 0.24–0.44) in the controls.

### Chronological change

**Fig. 2:**
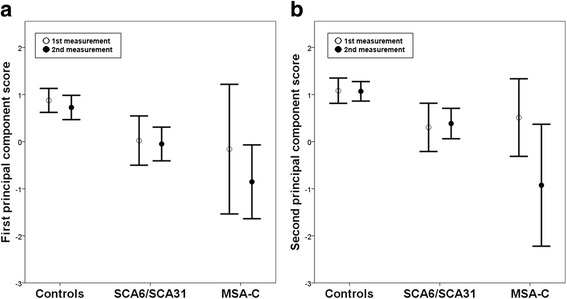
The chronological change of the first and second principal component scores. **a** The ﻿chang﻿e of the first ﻿principal component scores. **b** The change of the second principal component scores. The *bars* show the 95% confidence intervals. The number of subjects in each group was 18 in the controls, 2 in SCA6, 8 in SCA31, and 5 in MSA-C

Using the first and second PCSs, the chronological change with an interval of approximately 6 months was measured (Fig. 2). However, despite the fact that there were too few MSA-C patients to perform reliable statistical analysis, only the second PCS tended to decline in MSA-C patients (first PCS: *t* = 1.849, *p* = 0.138 and second PCS: *t* = 2.492, *p* = 0.067), whereas the changes of both the first and second PCSs were not apparent, not only in the controls (first PCS: *t* = 1.654, *p* = 0.116 and second PCS: *t* = 0.086, *p* = 0.933) but also in the SCA6/SCA31 patients (first PCS: *t* = 0.603, *p* = 0.562 and second PCS: *t* = −0.646, *p* = 0.535).

## Discussion

As PCA can yield orthogonal factors that account for the variance of data, it is used to divide multivariable data into several components. PCA has also been proven useful to resolve gait into its component elements [12, 13, 17], which were named Pace, Rhythm, Asymmetry, and Variability [12, 17]. Not all of the parameters in this study were the same as those used in previous reports [12, 17], but they were basically interchangeable considering the physiological meaning of each parameter. As for the results of PCA in the controls, it was not difficult to interpret the meanings of PC2, 3, and 4. Cadence had very high factor loading in PC2. In the same manner, only step symmetry was relevant in PC3, and step regularity was relevant in PC4. Thus, it was reasonable that PC2, 3, and 4 were named Rhythm, Asymmetry, and Variability, respectively. In PC1, velocity, step length, and RMSs in each direction were relevant. RMS is closely correlated with gait velocity in normal gait [5, 15]; therefore, it is reasonable that velocity and RMSs in each direction were contained in the same component. As a higher RMS value is caused by a faster gait velocity [5], gait velocity was more predominant in PC1 than RMS, supporting our contention that PC1 could be named Pace.

The results of PCA in the patients were considerably different from those of the controls. In the patients, the factor loading values of cadence, velocity, step length, and RMS were relevant to PC1. This means that PC1 was not able to differentiate the Pace factor from the Rhythm factor in the patients. In PC2, the difference between both groups was much more evident. To understand the meaning of PC2 in the patients more clearly, we calculated the second PCS for each subject. The second PCS was significantly different between the patients and controls, and was significantly correlated with disease duration and the SARA score of gait in the patients. The second PCS was also different between the subgroups divided according to the SARA score of gait. Hence, it is reasonable to suppose that PC2 in the patients represented the main component of ataxic gait.

RMS increases with the square of gait velocity in normal gait [5, 15]; thus, there should be a positive correlation between RMS and gait velocity in all directions. In fact, the factor loading values of PC1 in the controls showed a positive correlation between RMS and gait velocity. However, the factor loading values of PC2 in the patients demonstrated a negative correlation between RMS in ML and gait velocity. In addition, the factor loading values of PC2 indicated that RMS in ML was more relevant than that in the other directions when calculating the second PCS. As the second PCS had a significant negative correlation with the SARA score and disease duration, a smaller second PCS represented a higher severity of ataxic gait. From the results, PCA showed that a short step, low step regularity and symmetry, and a high degree of body sway in ML were characteristics of ataxic gait.

We considered that the second PCS would be effective for the assessment of chronological changes in the subjects. It is noteworthy that the range of the second PCS was very narrow, and there was no significant chronological change in the controls. Due to the small sample size, the chronological change of the second PCS could not be examined sufficiently in the patients. However, it was likely that the degree of the chronological change of the PCS would be different among the disease subtypes. In particular, the score change in the MSA-C patients, but not the SCA6/SCA31 patients, was relatively large.

A triaxial accelerometer is an objective and reliable tool to study standing and gait [6, 18]. Although the data obtained using a triaxial accelerometer are considered to be highly reliable, the reliability of new parameters calculated from the acceleration data needed to be validated. For this purpose, the intra-rater reliability of each gait parameter was assessed using ICC (1, 1). The ICC (1, 1) values of velocity, step length, and RMS in each direction were greater than 0.8 in the patients and controls. The ICC (1, 1) value of RMS in this study was approximately the same as that in a previous report [17]. We considered that the reliability of the instrument we used was demonstrated by this result. The ICC (1, 1) value of cadence in the controls was also similar to that in a previous report [19], but it was relatively low in the patients. This difference is due to gait disturbance in the patients. As for step symmetry, there was no significant difference between both groups. In addition, there was no significant correlation between step symmetry and disease duration (AP direction: *ρ* = −0.147: *p* = 0.260 and VT direction: *ρ* = −0.229: *p* = 0.076). These results indicate that step asymmetry was not very conspicuous in the patients with SCA and MSA-C at the stage at which they could stand and walk unaided.

SARA is used widely to assess the severity of ataxia in clinical neurology settings. However, SARA is an ordinal scale and the change of the total SARA score was approximately 1–2 per year in several subtypes of SCA [20–22]. These are the main reasons why SARA does not have sufficient sensitivity for the assessment of ataxic gait in SCA or MSA-C patients. Disease-modifying therapies are still under investigation for SCA or MSA-C, but short-term intensive coordinated rehabilitation has been shown to be effective for ataxia patients [23, 24]. Considering clinical trials of upcoming disease-modifying drugs or of robotics-assisted rehabilitation, additional quantitative methods are needed to investigate short-term (~1 year) changes in the severity of ataxic gait. Our findings indicated that the second PCS is a continuous value reflecting the characteristics of ataxic gait in SCA and MSA-C patients. PCA has been used previously to reveal some of the characteristics of pathological gait not only in neurodegenerative disorders but also in patients with hip fracture or spinal cord injury [25, 26]. Simplified gait parameters such as walking speed or walking distance are not sufficiently sensitive to assess the severity of pathological gait in such conditions, and PCA provides new walking paradigms beyond a simple gait parameter [26]. To the best of our knowledge, this study is the first to apply PCA to gait analysis in SCA or MSA-C patients, and we found that PCA clearly detected the characteristics of ataxic gait. We propose that the second PCS can help us assess ataxic gait more objectively and quantitatively than the available methods.

## Conclusions

PCA of gait parameters revealed the main component of ataxic gait. This component was characterized as low gait velocity, short step, low step regularity and symmetry, and a high degree of body sway in the medio-lateral direction. The PCS of the main component was significantly different between the patients and controls, and it was well correlated with disease duration and the SARA score of gait in the patients. Although more detailed validation is needed in future studies regarding how applicable it is to assess the severity of ataxic gait, the PCS will provide a novel quantitative biomarker to assess the severity of ataxia.

## Abbreviations

AC: Auto-correlation coefficient
ANOVA: Analysis of variance
AP: Anterior-posterior direction
ICC: Intra-class correlation coefficient
ML: Medio-lateral direction
MSA: Multiple system atrophy
MSA-C: Multiple system atrophy with predominant cerebellar ataxia
PC: Principal component
PCA: Principal component analysis
PCS: Principal component score
RMS: Root mean square
SARA: Scale for the Assessment and Rating of Ataxia
SCA: Spinocerebellar ataxia
SCA31: Spinocerebellar ataxia type 31
SCA6: Spinocerebellar ataxia type 6
VT: Vertical direction
